# 901. Evaluation of Azithromycin Usage Following a Pharmacist Discontinuation Protocol

**DOI:** 10.1093/ofid/ofac492.746

**Published:** 2022-12-15

**Authors:** Kelly M Percival, Patrick M Kinn, Lukasz Weiner, Dilek Ince

**Affiliations:** University of Iowa Hospitals & Clinics, Iowa City, Iowa; University of Iowa Hospitals & Clinics, Iowa City, Iowa; University of Iowa Hospitals & Clinics, Iowa City, Iowa; University of Iowa Hospitals & Clinics, Iowa City, Iowa

## Abstract

**Background:**

Azithromycin usage along with a beta-lactam is common in hospitalized patients with community-acquired pneumonia (CAP). The total recommended dosage is 1500mg as either 500mg on day one followed by 250mg daily for 4 days or 500mg daily for 3 days. It was observed that total doses of >1500mg were frequently administered at our institution. Institutional empiric treatment guidelines for CAP were updated and educational sessions were given for medicine providers in November 2019 to emphasize a total dose of 1500mg. This was further followed in April 2020 by the creation of a pharmacist collaborative practice agreement (CPA) to allow any unit-based clinical pharmacist to discontinue azithromycin for CAP after administration of a sum total of 1500mg.

**Methods:**

A retrospective quasi experimental study was performed to evaluate the impact of implementing a pharmacist CPA for discontinuation of azithromycin for CAP. The primary objective was to compare the proportion of patients receiving greater than 1500mg of azithromycin during the pre-implementation period of April 2018-March 2020 to the post-implementation period of April 2020-March 2022. Pre- and post-implementation period rates were compared using a Chi-square test. Secondarily, utilization of CPA as the discontinuation method was evaluated.

**Results:**

A total of 8,373 patients were included (5123 pre and 3250 post) in the analysis. The proportion of patients receiving >1500mg of azithromycin decreased from 29.32% pre-implementation to 20.22% post-implementation (P< 0.001). There was a decrease seen immediately after implementation that has been maintained over time, as shown in figure 1. The CPA was utilized for discontinuation in a minority of patients during the post-implementation period and the majority discontinued by pharmacists were per verbal order.

Percentage of Patients Receiving >1500mg Azithromycin

**Figure:**
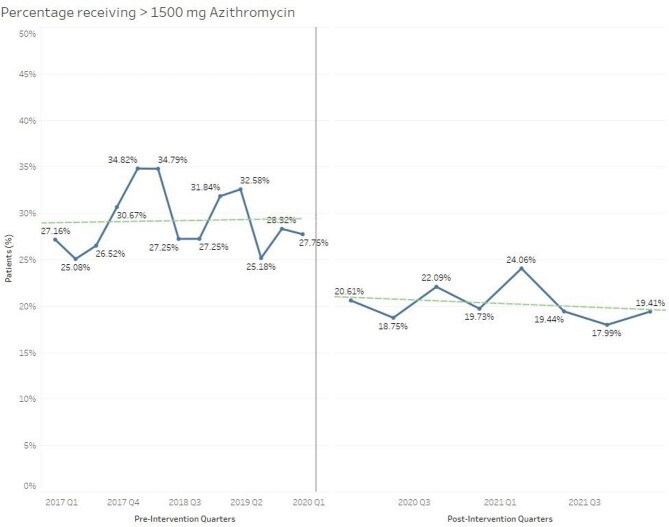

**Conclusion:**

Implementation of a pharmacist driven CPA for discontinuation of azithromycin for CAP reduced excessive azithromycin usage with minimal time burden for the AMS team. To further meet the goal of decreasing excessive azithromycin usage additional interventions are needed, and low utilization of the CPA should be further evaluated.

**Disclosures:**

**Kelly M. Percival, PharmD**, Gilead Sciences Inc: Advisor/Consultant **Patrick M. Kinn, PharmD, MPH**, Gilead Sciences: Advisor/Consultant **Dilek Ince, MD**, Evergreen: Member of data monitoring board|Gilead Sciences: Grant/Research Support|Leidos: Grant/Research Support|Moderna: Grant/Research Support.

